# The Piezoresistive Highly Elastic Sensor Based on Carbon Nanotubes for the Detection of Breath

**DOI:** 10.3390/polym12030713

**Published:** 2020-03-23

**Authors:** Romana Daňová, Robert Olejnik, Petr Slobodian, Jiri Matyas

**Affiliations:** Centre of Polymer Systems, University Institute, Tomas Bata University in Zlin, 760 01 Zlin, Czech Republic; olejnik@utb.cz (R.O.); slobodian@utb.cz (P.S.); matyas@utb.cz (J.M.)

**Keywords:** elastic sensor, carbon nanotubes, wearable electronics, monitoring of breathing, strain sensor, polymer composite, CNTs

## Abstract

Wearable electronic sensor was prepared on a light and flexible substrate. The breathing sensor has a broad assumption and great potential for portable devices in wearable technology. In the present work, the application of a flexible thermoplastic polyurethane/multiwalled carbon nanotubes (TPU/MWCNTs) strain sensor was demonstrated. This composite was prepared by a novel technique using a thermoplastic filtering membrane based on electrospinning technology. Aqueous dispersion of MWCNTs was filtered through membrane, dried and then welded directly on a T-shirt and encapsulated by a thin silicone layer. The sensing layer was also equipped by electrodes. A polymer composite sensor is capable of detecting a deformation by changing its electrical resistance. A T-shirt was capable of analyzing a type, frequency and intensity of human breathing. The sensitivity to the applied strain of the sensor was improved by the oxidation of MWCNTs by potassium permanganate (KMnO_4_) and also by subsequent application of the prestrain.

## 1. Introduction

Significant attention is devoted to wearable and flexible electronic technologies in the past decade. It is very important not to confuse the term “wearable technology” with “wearable device” or “smart device”. Wearable technology comes from describing the integration of electronics and computers into clothing or accessories that are comfortable on the body [1]. The research in an electronic textile has exposed to numeracy smart clothing applications for wearable electronic and healthcare monitoring. The base of success is an electrically conductive layer, which is flexible, stretchable, and lightweight [2].

Human breath is one of the basic natural bodily processes. Breathing can generally be divided into two basic types: diaphragm and thoracic. In the first case, the chest does not move, only the abdominal wall rises and falls. In the second type, in particular, the rib cage opens and contracts. [3].

The general trend of a healthy lifestyle is extended to areas that have the need to detect, record and analyze data from various human activities. There are already many advanced technologies that are also wearable but do not necessarily have integrated electronic or computing components. On the contrary, these belong to the range of smart textiles and clothing. Smart textiles are soft material with flexibility and drapability that are able to sense external conditions or stimulus, to respond and adapt to the behavior to them in an intelligent way. The stimulus that is detected by the smart textiles may have thermal, mechanical, chemical, electrical, magnetic and also optical properties. Similar formulations include intelligent, interactive, sensitive and adaptive. Smart textiles describe a novel category of textiles, which have the capability to detect and react to an external stimulus. In the current situation they can provide required information and help to master everyday life more effectively. They present a challenge in several fields of application such as health and sport. Among the first areas in which they appeared is military and health care [4,5]. In contrast, ultra-strong fire-resistant or breathing fabrics are not considered smart functionalized materials. In terms of the degree of intelligence, it is possible to classify intelligent textiles according to whether they can perform one or more of the following functions:

I. Passive smart textiles—ability to sense environmental conditions or an external element. The first generation of smart textiles that integrates sensors as conductive materials or optical fiber.
II. Active smart textiles—ability to respond after capture. The textiles consist of sensors and actuators as membranes, hydrogels and chromatic materials that provide the ability to sense and actuate or move a part of their environment.
III. Very smart textiles—ability to sense, react and adapt based on the learned experience from what it sensed and reacted to previously as thermo regulating clothing, space suits and health monitoring apparel for example [4,5].

Moreover, it is important to distinguish ‘intelligent’ clothing in terms of the degree of integration that relates to the extent to which the part that performs the intelligent function is embedded into the textile. At the lowest degree of integration, the smart material is attached to the surface of textile. In the second generation of intelligence, the material is integrated directly into the textile structure, for example, weaving or knitting conductive yarns to form a textile pressure sensor. The third generation of intelligence, which is the highest degree of integration is manifested innately as part of a yarn or fiber. It is thus able to be integrated much more discreetly without compromising user comfort. [1].

The term of electrically conductive fabrics are fabrics that can conduct electricity. They are designed for a wide range of textile fibber products woven from metal strands with very different specific electrical conductivity. This group includes conductive fibers, yarns, fabrics and finished products thereof [4].

One of the interesting and important things to be monitored is certainly the detection and monitoring of human breath. The frequency and intensity of respiration and the type of respiration are detected as a vertical distribution of breath parameters on the human body [6].

In addition, these new sensor technology materials have unique features such as high sensitivity, an ability to detect large-scale deformations and are flexible, lightweight and easy to manufacture. Moreover, in many cases they can be made multifunctional and have other utility properties. Sensitivity can also be successfully controlled. Their multifunctionality can be represented by the following properties: they can have thermoelectric properties, serve as a passive antenna element or a resistance heating element. Last but not least, they can be used to monitor the sensor fabrication process [7,8,9].

The first network of carbon nanotubes was made by Walters et al. in an attempt to vaporize graphite using a high energy laser, where the authors dispersed nanotubes into a liquid suspension and then filtered through fine filtration mesh. As a result, pure nanotubes stuck together to form a thin, free-standing, entangled structure that was later named Bucky Paper [9,10].

The aim of this article was a complex study of the electrical conductivity of multiwall carbon nanotube network both in the course of monotonic strain growth and also when loading/unloading cycles were imposed [8]. The multiwalled carbon nanotubes (MWCNTs) network was fixed on a polyurethane dog bond shape test specimen for the creep test. The current proposed solution of polymer composite highly elastic and elongation sensors brought a new generation of these deformation-electrically sensitive converters based on materials from the area of nanocomposite materials. These can be deformed in a wide range of deformations (chest deformation during breathing is in the range of units of percent to about 14%), then with high sensitivity, which can be significantly increased by chemical functionalization [11,12,13,14,15].

In the field of application, cooperation is underway, which leads to the prototype’s creation of a breath monitoring system including its own T-shirt integrated sensors, electronics with data transmission and subsequent visualization in a mobile application. In the future, it is possible to consider using this system for sports and fitness clothing, when the sensor will record biometric data, analyze this data for users, which can lead to learning and performing these activities better, it can lead to improved performance of athletes, and the daily activities of the individual. In the present paper the fabrication of flexible and stretchable (carbon nanotubes: CNTs) strain sensor was demonstrated, which in general is applied for human breath monitoring [7].

The article was focused on both the areas, wearable electronics and preparation of highly elastic piezoresistive sensors for the detection of tensile deformation based on an elastic polymer and carbon filler such as carbon nanotubes (CNTs). A highly elastic sensor for a T-shirt was overlaying by the active layer to avoid contact with surroundings and development of the collecting electrode system [9]. The commonly used metal wire strain gauge, which is normally produced in the form of a wire with precise geometry in the elastic film. The ration between resistivity change and deformation is called the gauge factor (GF). The commercial strain sensor has a GF of about 2–5 and an elastic deformation capability in unit %. The sensitivity factor is given by the ability of the wire and foil to react to the applied deformation [11].

## 2. Materials and Methods

The following raw materials and applied manufacturing procedures were used to achieve the desired functionality.

The materials were also analyzed by X-ray photoelectron spectroscopy (XPS) on TFA XPS Physical Electronics instrument (PHI-TFA, Physical Electronics Inc., Chanhassen, MN, USA) [16,17] at the base pressure in the chamber of about 6 × 10^−8^ Pa. The samples were excited with X-rays over a 400 µm spot area with a monochromatic Al K_α1,2_ radiations at 1486.6 eV. Photoelectrons were detected with a hemispherical analyzer positioned at an angle of 45° with respect to the normal to the sample surface. Survey-scan spectra were made at a pass energy of 187.85 eV, the energy step was 0.4 eV. Individual high-resolution spectra for C 1s were taken at a pass energy of 23.5 and 0.1 eV energy step. The concentration of elements was determined from survey spectra by MultiPak v7.3.1 software from Physical Electronics (Physical Electronics Inc., Chanhassen, MN, USA).

Purified multiwall carbon nanotubes (MWCNTs) were received from Sun Nanotech Co. (China). CNTs were synthesized by chemical vapor deposition method of acetylene (CVD). Acetylene was used as a precursor. MWCNTs has the following properties: a diameter of 10–30 nm, length of 1–10 μm, electrical resistivity of 0.12 Ωcm and purity more than 90%. By transmission electron microscopy (TEM, JEOL Ltd., Tokyo, Japan) the diameter of individual nanotubes was found to be between 10 and 60 nm and their lengths were from tens of micrometers up to 3 μm [14]. The maximum aspect ratio of the nanotubes was about 300 [13].

Non-woven polyurethane (PU) porous membranes for MWCNTs dispersion filtration were prepared by electrostatic spinning from a PU solution. Electrostatic spinning from polyurethane dimethyl form amide/methyl isobutyl ketone (DMF/MIBK, 1:3) solution was performed in cooperation with the SPUR a.s. company of the Czech Republic in Zlin. The conditions of electrospinning were as follows: a PU concentration of 16 wt %. Electrical conductivity of the solution was adjusted to 20 μS/cm using sodium chloride, an electric voltage of 75 kV (Matsusada DC power supply, Matsusada, Shiga-ken, Japan) was applied. Temperature of 20–25 °C, and a relative humidity of 25%–35% was controlled during the process (for a detailed schematic of experimental part, Kimmer et al. [12]). Thermal properties were measured by differential scanning calorimetry (DSC, Mettler Toledo, Columbus, OH, USA) on a Mettler Toledo DSC1 STAR System (Mettler Toledo, Columbus, OH, USA). The measurements were performed under nitrogen atmosphere. The first heating was set to erase the thermal history from 25 to 180 °C (heating rate 10 °C·min^−1^) with a 5 min isotherm, then cooling from 180 to −60 °C (cooling rate 10 °C·min^−1^), the isotherm at −60 °C for 5 min. The second heating cycle was from −60 to 250 °C (10 °C·min^−1^) and was intended for analysis of the test material.

A thermoplastic polyurethane (TPU) Desmopan DP 385S (Bayer MaterialScience, Leverkusen, Berlin, Germany). The ultimate strength of TPU of 48.9 MPa, with the strain at break 442.2% and density of 1.20 g/cm^3^ were specified by the supplier. Used TPU is high elastics elastomeric polyurethane with an ultimate strength up to 400%. It serves as an elastic base for the MWCNT sensory layer. It is thus an integral part of the sensor, which consists of these two functional layers. By this process a high flexible and elastic sensor is prepared to be capable to measure deformations of a large extent. It is also important that the polymer is thermoplastic. The filter membrane then melts in the preparation process and becomes an adhesive layer between the two components. In conclusion, other polymeric elastomeric matrices can also be used.

KMnO_4_ oxidation: Oxidized MWCNTs were prepared in a glass reactor with a reflux condenser filled with 250 mL of 0.5 M H_2_SO_4_, into which 5 g of potassium permanganate (KMnO_4_) as an oxidizing agent and 2 g of MWCNTs were added. The dispersion was sonicated at 85 °C for 15 h using thermostatic ultrasonic bath (Bandelin Electronic DT 103H, Berlin, Germany). The dispersion was filtered, and then MWCNTs were washed with concentrated HCl to remove MnO_2_ and then washed with water until the system attained a pH of 7.

Aqueous dispersion of MWCNTs was prepared by sonication in an apparatus UP 400S from Dr. Hielscher GmbH (ultrasonic horn S7, amplitude 88 μm, power 300 W and frequency 24 kHz, Stuttgard, Germany) for 15 min at room temperature. The nanotube concentration in the suspension was 0.3 wt %. Dispersion also contained surfactants, namely, sodium dodecyl sulphate and 1-pentanol with a concentration of 0.1 and 0.14 M, respectively. Moreover, NaOH aqueous solution was added to adjust the pH to 10. For making an entangled MWCNTs network, a porous polyurethane membrane and a vacuum filtration method was used. About 30 mL of homogenized dispersion was filtered through a funnel of diameter 90 mm. The prepared MWCNTs network was washed several times with deionized 60 °C hot water, afterwards by methanol in situ and dried between two filtration papers for 24 h.

The networks of MWCNTs (pure) and oxidized MWCNTs (KMnO_4_) were selected for the creep test. The composite stripes 55 mm × 10 mm (L × W) were welded onto the polyurethane bodies and gradually loaded from 0.167 to 1.066 MPa.

A composite stripe of 55 mm × 10 mm with a thickness of 0.08 mm and weight was approximately 2.57 mg was directly welded onto a commercial sport T-shirt to monitor human breathing. The electrical resistance change was measured along the specimen length by the two-point technique using Multiplex datalogger 3498A. The electrodes for a two-point electrical resistance measurement were prepared from very thin Cu wires emailed to sensors with Ag conductive lacquer. The time for hardening of the Ag conductive lacquer after application on the sensor was 2 h. This mentioned technique does not affect the elasticity of the used T-shirt. The electrical resistance measurements as dependence on time were performed during breath and exhale cycles of breathing. The commercial sport T-shirt was chosen from a Czech sports brand (Moira CZ a.s., Strakonice, Czech Republic), Moira, with a composition of 97% polypropylene from the brand Moira and 3% elastane. The T-shirt has to be tight in the entire upper half of the body therefore the size XS–S was chosen. The polyester interlayer strip is necessary for ironing the sensor o the T-shirt.

Two components silicone rubber GMS 2628 from Dawex Chemical s.r.o. (Zlin, Czech Republic) having a hardness score of 26–28 A and with high elasticity was also used to cover the final sensor.

The change in electrical resistance of the MWCNTs network in breath/exhale cycles was measured lengthwise by the two-point method using Multiplex datalogger 34980A (Keysight technologies, Santa Rosa, CA, USA) connected to a PC with a sampling frequency of 10 Hz.

Micrographs using scanning electron microscopy (FEI Nova NanoSEM 450, (FEI company, Hillsboro, OR, USA) were used to observed MWCNTs (pure) and MWCNTs (KMnO_4_) samples and also a semi product of filtration and final TPU/MWCNTs composites. The samples were fixed with adhesive tape on the aluminum stub.

## 3. Results

**Figure 1:**
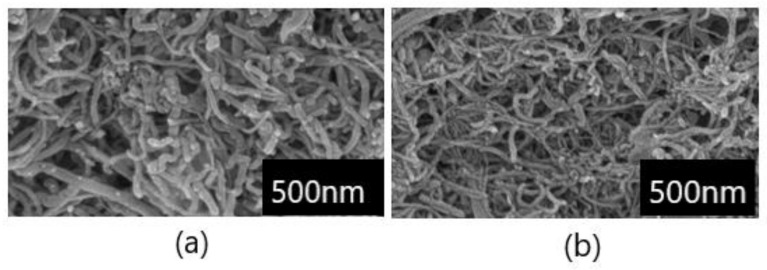
The scanning electron microscope (SEM) micrograph of carbon nanotubes network. The upper surface of entangled carbon nanotube network prepared by the filtering method from multiwalled carbon nanotubes (MWCNTs; pure) and oxidized MWCNTs (KMnO_4_). (**a**) SEM micrograph of the MWCNTs (pure). (**b**) SEM micrograph of MWCNTs (KMnO_4_).

Two types of MWCNTs were used for this article and tested as pure and oxidized, respectively. Based on the results of the creep test, a sample of oxidized MWCNTs (KMnO_4_) was selected and with values of MWCNTs (pure) was compared. The network of MWCNT (pure) was composed of long entangled tubes (Figure 1A) while the network of MWCNTs (KMnO_4_) was shortened due to previous oxidation (Figure 1B). Therefore, the number of contacts was maximized, resulting in high sensor sensitivity to strain changes.

**Figure 2:**
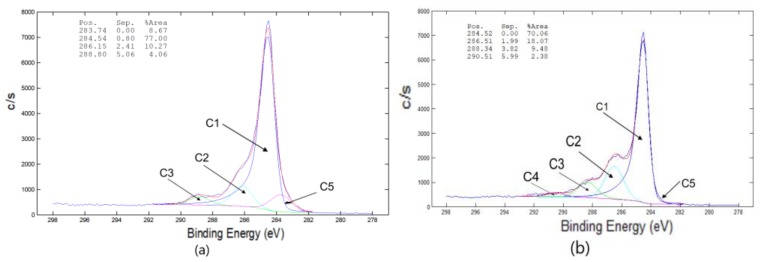
X-ray photoelectron spectroscopy (XPS) on CNT/PU and CNT/PU KMnO_4_. (**a**) Curve fitting of the C 1s carbon spectrum of the MWCNTs (pure). (**b**) Curve fitting of the C 1s carbon spectrum of MWCNTs (KMnO_4_).

The main binding energy peak (284.5 eV) in the XPS spectra of MWCNT was assigned to the C1s-sp^2^, while the other ones were assigned to C–O (286.15 eV), C=O (287.1 eV), O–C=O (288.8-289 eV) and C1s-π-π* (291.1–291.5 eV). According to our XPS results of MWCNT the total oxygen content was determined to be 18.8 at % for pure MWCNT and 21.4 for the oxidized one. The sp^3^/sp^2^ carbon ratios were 2.50 and 1.69 for pure MWCNT and KMnO_4_ oxidized ones (Figure 2), respectively [7].

The resistivities of the network structures were measured to be 0.084 ± 0.003 Ωcm for the network fabricated from pure MWCNTs and 0.156 ± 0.003 Ωcm for the network made of KMnO_4_ oxidized tubes [18].

Porosities of two principal networks were calculated to be 0.67 and 0.56, for pure and oxidized network respectively [18].

The principle of the sensing is in the reduction of intertube contacts and the creating of cracks. When the composites are elongated, the number of contacts decreases leading to macroscopic resistance of the sensory layer. Oppositely, when the composite relaxed, the number of contacts increases, which leads to a reversible decrease of the composite resistance. As was demonstrated elsewhere [19], oxidation leads to the formation of oxygenated functional groups detached to the CNT surface causing an increase of the contact resistance in CNT junctions. It finally increases sensor sensitivity to the strain when a sharper resistance change is observed when deformed.

**Figure 3:**
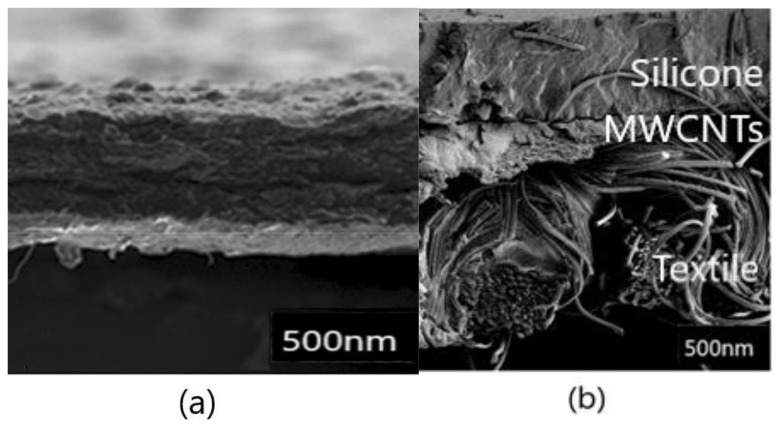
(**a**) The cross-section of a MWCNT film (above) and supporting TPU filtering membrane (underneath). (**b**) MWCNT composite cross-section after the melt welding.

Morphology of the prepared composite acting as a strain sensor can be demonstrated by Figure 3. In principle it is the composite created by the three layers as a commercial T-shirt, a CNT/TPU interface layer and two-component silicone rubber [19].

**Figure 4:**
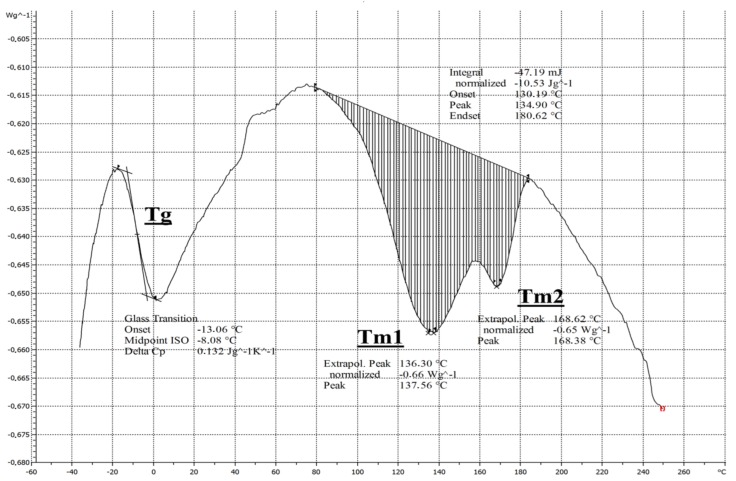
Differential scanning calorimetry (DSC) of a non-woven polyurethane membrane.

Figure 4 characterizes the DSC analysis of the non-woven thermoplastic PUR membrane. A glass transition (*T_g_*) of the elastomeric component was seen, *T_g_* = −8.08 °C. It is also possible to follow two successive areas melting of the material (endothermic process) *T*_m1_ = 137.56 °C and *T*_m2_ = 168.38 °C.

**Figure 5:**
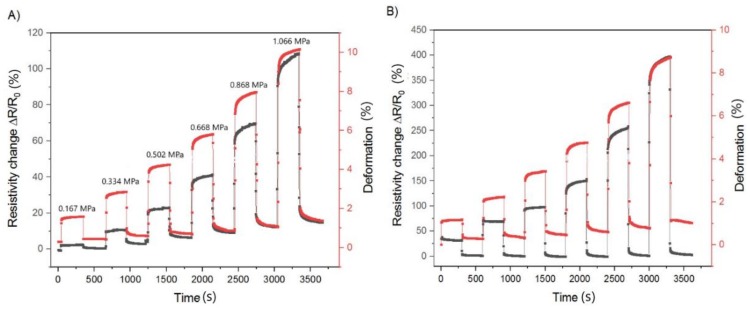
The comparison of the relative resistance change vs. the deformation for the MWCNTs (pure) (**A**) and MWCNTs (KMnO_4_) (**B**) sensor in six extension/relaxation cycles induced by the tensile stress (from 0.167 to 1.066 MPa). The tensile stresses are the same for both measurements. The deformation is denoted by red circles, the relative resistance changes by black circles.

In the first case, the sensor of MWCNTs (pure) and oxidized MWCNTs (KMnO_4_) were deformed by a tensile stress from 0.167 to 1.066 MPa in six extension/relaxation cycles and were compared. The results are shown in Figure 5 as a percentage change in the relative resistance,

ΔR/R_0_ = (R − R_0_)/R_0_ (1)

where R_0_ is the electrical resistance of the measured sample before the first elongation and R is the resistance during elongation, strain is the relative change in specimen length.

The elongation periods when the stress was in range from 0.167 to 1.066 MPa, and relaxation when the load was removed was 300 s (Figure 5). The maximum resistance changes of MWCNTs (pure) were 110% and the deformation was 10%. The maximum resistance changes of MWCNTs (KMnO_4_) were 400% and the deformation was 9%, which implied a fourfold increase in sensitivity.

Further, the sensitivity to applied strain, deformation ε, is defined by the gauge factor (*GF*)

GF = (ΔR/R_0_)/ε (2)

**Figure 6:**
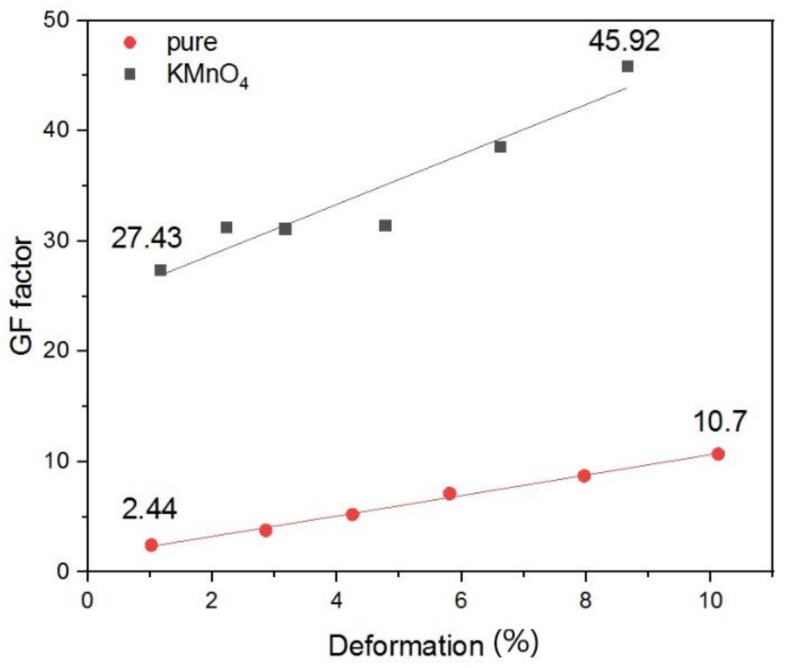
Dependence of gauge factor (GF) with increasing deformation for MWCNTs (pure) and oxidized MWCNTs (KMnO_4_) sensors.

Strain represents a relative change in specimen length, as the ratio between the change of specimen length, *ΔL*, relative to initial length, *L_0_*, *ε = ΔL/L_0_*. Figure 6 shows the dependence of the gauge factor *GF* on deformation *ε*.

The issue was the creep test, which means loading by constant extension stress followed by measurement of the specimen deformation and resistance in time. After 5 min of loading, it was followed by relaxation in another 5 min in the off loaded state when the reversible creep occurred in time. The specimen was then loaded with an appropriate higher stress. Finally, there were six consecutive loading/unloading cycles. Elongation of the specimen led to an increase in composite macroscopic resistance and resistance reversible decrease when the specimen was in an off load state and the deformation relaxed. In general, the sensor is then sensitive to deformation and this response is reversible. The measured value of *GF* reached for the example value of 11 for MWCNTs (pure) and of 46 for MWCNTs (KMnO_4_) at an applied deformation 10% (Figure 6). The principle of sensing is in the formation of a microcrack, which decreases the quantity of intertube contacts, which leads to the macroscopic resistance increase. When the composite relaxes, the microcrack close leading to a reversible resistance decrease. Oxidation incorporates more functional oxygenated groups chemically detached to the CNTs surface leading to a sharper increase of resistance during deformation and a more sensitive sensor to strain. Prestrain leads to the formation of a crack, which is easier opened in the next cycles again leading to higher sensor sensitivity [19].

**Figure 7:**
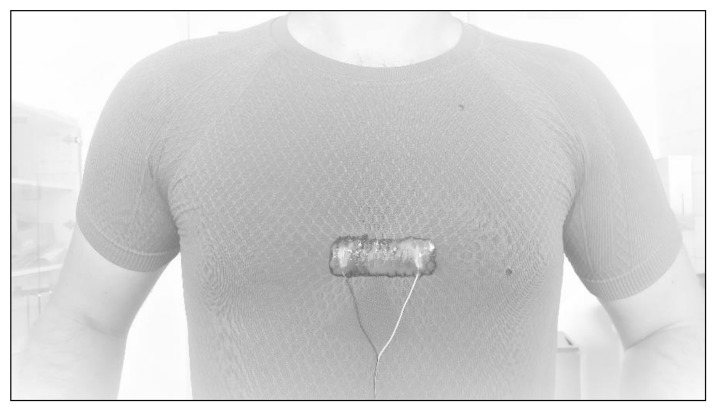
Sensor based of MWCNT (KMnO_4_) fixed on a sports T-shirt for practical application of a human breath monitoring.

**Figure 8:**
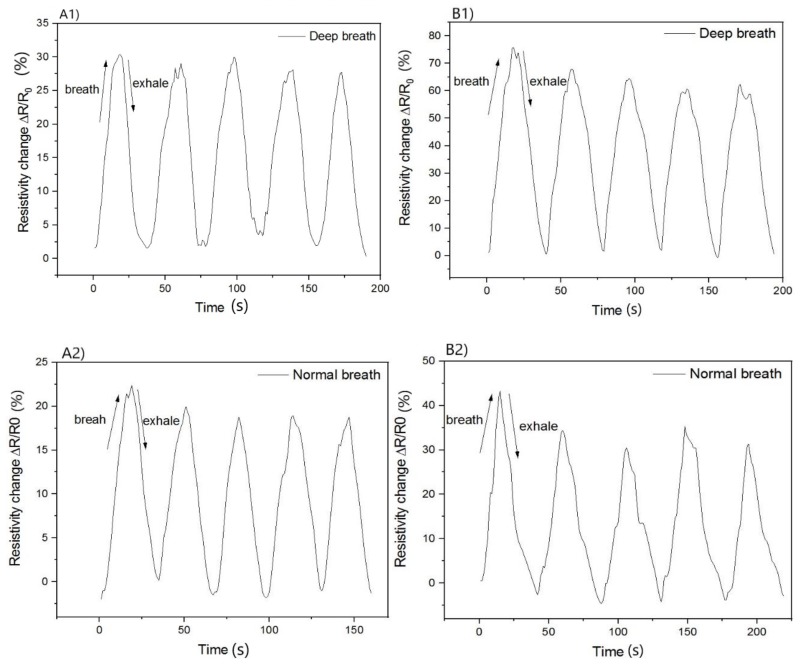
Response to a change in relative resistance, ΔR/R_0_, of carbon nanotube sensors (MWCNTs/KMnO_4_) integrated into the T-shirt to monitor human breath. The comparison of deep breath and normal breath for both volunteers (**A1**,**A2**,**B1**,**B2**).

The next section describes the application of a strain gauge to monitor human breath in two volunteers. Subsequently, different types of breathing were observed, namely deep and normal breathing. As shown in Figure 7, this integrated MWCNTs (KMnO_4_) sensor was fixed on a T-shirt. Two volunteers A and B were selected to test normal and deep breathing. The maximum deformation of the chest the volunteer (A) was 5% with deep breathing and the change in electrical resistance was 30%. In the volunteer (B), a maximum chest circumference deformation was 7% for deep breathing and a change in electrical resistance was 65% (Figure 8).

The response of the sensor to electrical resistance was therefore very sensitive to deformation. The sensor was reversible and was able to detect breathing in real time.

The stabilizing effect on the resistance extension cycles and the residual normalized resistance change was constant after approximately five cycles. That is, during the first deformations, the network of nanotubes acquired a structure that remained more or less the same regardless of the number of deformation cycles. This mechanical stabilization is advantageous for using the sensor as an elongation sensing element.

## 4. Conclusions

A highly elastic deformable and piezoresistive sensor composed of a network of an electrically conductive entangled carbon nanotubes network built into the elastic commercial T-shirt and encapsulated by elastic silicone was introduced. This material was sensitive enough to be used to monitor human breath. It did not influence any human activity. Respiratory characteristics in two volunteers were compared. The monitoring of breath worked on the principle for the sensors electrical resistance change. Possible application of the sensor in an elastic T-shirt can not only be for athletes, but also for newborns with serious sleep disorders such as sleep apnoea, which can be used to prevent premature death.
